# Exploring senior managers’ experiences preparedness to respond to COVID-19: a qualitative study in Iran

**DOI:** 10.1186/s12913-023-09764-2

**Published:** 2023-07-20

**Authors:** Hojatolah Najafi, Zahra Kavosi, Mahnaz Rakhshan, Jalal Karimi, Milad Ahmadi Marzale, Mostafa Bijani, Mahmoudreza Peyravi

**Affiliations:** 1 Department of Health in Disasters and Emergencies, Health Human Resources Center, School of Health Management and Information Sciences, Shiraz University of Medical Sciences, Shiraz, Iran; 2 Health Human Resources Center, School of Health Management and Information Sciences, Shiraz University of Medical Sciences, Shiraz, Iran; 3 Department of Nursing, School of Nursing and Midwifery, Shiraz University of Medical Sciences, Shiraz, Iran; 4 Department of Infection Disease, Faculty of Medicine, Fasa University of Medical Science, Fasa, Iran; 5 Department of Medical Surgical Nursing, Fasa University of Medical Sciences, Fasa, Iran

**Keywords:** Emerging respiratory viruses, Health system, Preparedness, Qualitative study

## Abstract

**Background:**

Epidemics caused by emerging respiratory viruses are challenging for the health system of most societies, and preparedness of the health system in responding to such epidemics is important. Therefore, the aim of this study was identifying different fields and key issues of the senior managers’ experiences preparedness to respond to the COVID-19 epidemic from the Iranian senior managers’ point of view.

**Methods:**

This is a qualitative descriptive study. Eighteen in-depth and semi-structured individual interviews were conducted for data collection. For this purpose, 18 senior managers with work experience in managing the COVID-19 crisis were enrolled in the study using purposive sampling. The collected data were analyzed according to Graneheim and Lundman’s approach.

**Results:**

Analysis of the data resulted in the emergence of five themes and twelve sub-themes. The main themes and sub-themes included: (1) capacity improvement consisting of performance improvement and logistic improvement; (2) resource and infrastructure management including supply and support of human resources, infrastructure improvement, and supply of equipment; (3) an increase in epidemiology capacity including epidemiology improvement and emerging disease surveillance; (4) application of the principles of disaster and emergency management including intra- and extra-organizational interaction management, disaster risk management, and data management; and (5) society resilience increase including improving adaptation skill and maintaining health and social participation.

**Conclusion:**

The results of this study present the key issues for the management of future emergency situations. Health system managers and policymakers in Iran and other countries should be aware of these key issues and apply them in practice to prepare the health systems to respond to next outbreaks. Indeed, the study results can help policymakers and health system managers to plan to achieve acceptable preparedness for the management of such outbreaks.

## Introduction

Emerging infectious diseases and their threats to global health are increasing; among the emerging infections, respiratory pathogens, which are common causes of acute events in a large number of patients, posed a serious concern for the health system [1]. In this situation, the pressure on the health care delivery system is at its maximum level; health care needs have remarkably risen, and the care resources are scarce; as a result, the care delivery process has become an emergency. These diseases can affect the global health system and cause serious challenges for this system [2, 3].

The most recent emerging infectious disease is COVID-19, which has infected all countries and become a global emergency. Even countries that are economically and healthily capable could not escape from this virus and their health system has faced a major challenge [4, 5]. In other words, COVID-19 pandemic is like a stress test for all aspects of life, especially health, and revealed the weaknesses of the health system in terms of capacity and structure [6]. For example, a study conducted in a hospital in Italy showed severe contamination of the hospital, allocation of more beds to COVID-19 patients, lack of intensive care beds, insufficient logistic and treatment services, even lack of resuscitation of the elderly, and lack of medicine, ventilators, oxygen and personal protective equipment at the onset of the COVID-19 pandemic [7].

Iranian health system has also been struggling with this pandemic and experienced various challenges. Cultural challenges, lack of human resources, ad lack of personal protective equipment and diagnostic and treatment facilities have been reported as important challenges to the health system during the COVID-19 pandemic [8].

In fact, the spread of newly emerging viruses and their subsequent challenges indicate the need for the health system preparedness to respond to these viruses. On the other hand, according to the United Nations office for Disaster Risk Reduction (UNDRR), the current approach to disaster preparedness is the All-Hazards Approach (AHA). However, an increase in hazards and development of studies in this field indicate the fact that this approach has several shortcomings and is not effective in planning for preparedness for all risks. In particular, the occurrence of epidemics in recent years, especially the COVID-19 pandemic, showed that disasters caused by epidemics were unique and required their own planning to respond [9]. Therefore, to plan for the health system preparedness for these epidemics, the fields in which health systems need to be prepared should be specifically investigated and known. On the other hand, investigating and knowing these fields need conducting qualitative studies [10]. According to our search, no qualitative study was found based on the cultural background, management, beliefs, and values ​​of the Iranian society in this field. So, it is important to conduct qualitative studies to search, describe and gain a deep insight into managerial factors, and investigate knowledge, culture, beliefs, values, experiences ​​ related to newly emerging viruses management in society.

Additionally, an understanding of senior managers’ experiences on preparedness to respond to COVID-19 is important because it will help the authorities to identify the factors that improve the quality of management. Therefore, the aim of the present study was to explore the experiences of senior manager’s preparedness to respond to COVID-19 in Iran.

## Methods

This is a descriptive qualitative study conducted using content analysis. Conventional qualitative content analysis is employed when little data is available about the desired concept. Since it is important to know experiences of the health system managers and experts about the fields of the health system preparedness in responding to emerging respiratory viruses, this approach was adopted taking into account the deep and comprehensive understanding of their experiences in this field as well as the cultural, social, and political characteristics [11].

### Participants

The study group included senior managers and experts working in universities of medical sciences, hospitals, and health centers affiliated with universities of medical sciences in Iran. Eighteen individuals who met the study inclusion criteria were selected from medical sciences universities in different parts of Iran in the north, south, east, and center and participated in the present study. In addition, based on the objective of the study, purposive sampling was used [12].

The study inclusion criteria were at least a bachelor’s degree in one of medical sciences and health (nursing, health, management of health care services) or general medicine sub-fields; at least one year of work experience in the management of emerging respiratory viral diseases or education and research such as doctorate in health in disasters and emergencies; willingness to participate in the study; deep, rich and sufficient information about the desired concept; and ability to provide this information. The study exclusion criteria were unwillingness to continue the interview and participate in the research and lack of participation in the interview sessions.

### Data collection and analysis

After obtaining the permission of the Ethics Committee and an introduction letter from Shiraz University of Medical Sciences, data collection was done. For this purpose, twenty individual and semi-structured interviews were conducted with 18 people who met the study inclusion criteria from Sep. 23, 2021 to Dec. 21, 2021. Thirty two senior managers’ and experts working in universities of medical sciences, hospitals, and health networks affiliated with universities of medical sciences were invited to participate in the study, but 14 subjects refused to participate due to busy schedule, intensive shifts, infection with COVID-19, and willingness to spend time with their families. Therefore, the participants consisted of 18 senior managers’ who were selected via purposeful sampling.

Purposeful sampling is one of the frequently used methods in qualitative studies. In this type of sampling, the subjects are selected by the researcher. The subjects who are selected have rich and in-depth knowledge and experience of the concept under the study and can provide a detailed insight into it [11]. Thus, the researchers selected individuals with extensive experience or knowledge of the subject of the study. After consulting the managers and experts working in universities of medical sciences, the researchers selected one of the personnel who could interact better and provide rich information for the initial interview. Then, the first participant was asked to name a colleague who had rich knowledge and experience of the subject under the study. In this manner, based on the information provided by the managers and participants chosen earlier, more participants who had a good understanding of the subject of the study were selected. Sampling continued until we reached data saturation. Data saturation is achieved when no new data could be collected and no new categories or sub-categories could be extracted [12]. In this study, data saturation was reached after 18 interviews, and two further interviews were done to ensure that no new data was available.

The time and place of the interviews were coordinated based on the participants’ desires. The first author (HN), who had led numerous qualitative studies, conducted the interviews. Interviews started with the introduction of the researcher and then general questions such as: “Can you describe your one-day work experience in crisis management of emerging diseases such as COVID-19?“ and then continued with specific questions including “Based on your experience, how is health system preparedness defined in response to emerging respiratory viral diseases?“ and “What strategies do you suggest to improve the preparedness of the health system in the face of emerging respiratory viral diseases?“ To increase the clarity of information, we asked probing questions such as “Can you explain more?“, “What do you mean?“, and “Can you give an example?“ The process of interviews was followed according to the main objective of this study. The interviews lasted about 40–60 minutes. After finishing each interview, the corresponding author immediately listened to the interviews several times and then transcribed them on paper.

### Data analysis

Data were analyzed at the same time as data collection immediately after conducting each interview, and then the next interview was planned based on the results of the interviews done. They were analyzed by Granheim and Lundman’s (2004) conventional content analysis. For this purpose, with obvious and hidden content of the semantic units, the key points of the text were extracted in open code and manually. These codes were classified based on similarities and differences, and the process of abstraction continued until categories were extracted [13]. Immediately after each interview, the first author (HN) transcribed the recorded content and marked the paragraphs which were significant. After the meaning units were selected, each unit was assigned a code. Subsequently, the second author (ZK) examined the transcripts and verified the units and codes. Then, the homogeneous codes were merged and the categories were developed. To confirm the reliability of the codes, the researchers examined the categories and compared them to the initial data. Finally, in several meetings, the research team explored the categories and extracted the themes. The collected data were analyzed using MAXQDA v. 2007.

### Rigor (accuracy and trustworthiness of the research data)

To ensure the validity and reliability of qualitative data, we used Lincoln and Guba’s four criteria of credibility, dependability, confirmability, and transferability (2011). For credibility, to perform peer-checking, the researchers asked five experts to analyze and observe the process of data analysis and validate the codes and categories. Also, for data collection and analysis, prolonged engagement, comparative and continuous analysis, review of the participants, review of the colleagues, and writing notes were used. For dependability, an audit trial was used, which means that the data were provided to 4 researchers who were not related to the research and were external reviewers, who also had a similar understanding of the data. All stages of the research from the beginning to the end were explained in detail and the external reviewers performed the audit according to these documents. Also, the colleagues’ analyses and search for contradictory evidence were used for confirmability. Finally, for transferability, the research process for judging and evaluating others, sufficient number and diversity of the participants, and purposive sampling were described [14].

### Ethical considerations

All participants signed a written and informed consent to participate in this study. The present study was conducted in accordance with the principles of the Revised Declaration of Helsinki, a statement of ethical principles that guides physicians and other participants in medical research involving human subjects. The participants were assured of the anonymity and confidentiality of their information. Also, the study was approved by the Ethics Committee of Shiraz University of Medical Sciences in Iran, with the ethics code of IR.SUMS.NUMIMG.REC.1400.025.

## Results

A total of 18 managers and specialists participated in this study. Twelve participants (66.6%) were men and 6 (33.3%) were women. The mean age of the participants was 44.5 years, and the mean work experience was 16.66 years. The demographic and occupational information of the participants is given in Table 1. The views of the participants about the fields in which the health system should be ready to respond to newly emerging respiratory viruses led to the extraction of five themes and twelve sub-themes. The five main themes included clinical capacity improvement, resource and infrastructure management, epidemiology capacity increase, application of the principles of disaster and emergency management, and increase in the resilience of the society. The themes and sub-themes are shown in Table 2.

**Table 1:** General description of the participants

Participants	Work experience (year)	Job position and role in the epidemic	Education
P1	20	Coordinator between health system and external organizations during COVID-19 outbreak	PhD in Anatomical Sciences
P 2	17	ED head nurse during COVID-19 outbreak	Bachelor of Nursing
P3	21	EMS manager during H1N1 and COVID-19 outbreak	PhD in health in disasters and emergencies
P4	19	EMS manager during H1N1 and COVID-19 outbreak	PhD in health in disasters and emergencies
P5	30	Manager of health education and health promotion unit during H1N1 and COVID-19 out breaks	Master’s degree in social education in the health system
P6	9	Manager of infectious diseases unit during COVID-19 out break	Bachelor of Combating Diseases
P7	17	ED general practitioner during Covid-19 outbreak	General practitioner
P8	2	Expert in disasters and researcher in COVID-19 field	PhD in health in disasters and emergencies
P9	10	Medical manager of COVID-19 ward and member of COVID-19 management committee	Infectious disease specialist
P10	15	COVID-19 Ward head nurse	Bachelor of Nursing
P11	16	Hospital Manager	PhD in health in disasters and emergencies
P12	25	Member of the national staff to deal with COVID-19	PhD in health education and health promotion
P13	11	ICU COVID-19 head nurse	Bachelor of Nursing
P14	23	Dean of Medical Sciences University during COVID-19 and H1N1 outbreaks	Emergency medicine specialist
P15	9	Member of contagious diseases including COVID-19 research center	PhD in epidemiology
P16	12	COVID-19 center manager	Master’s degree in healthcare management
P17	14	Medical manager of ICU COVID-19	Internist
P18	30	Member of the national staff to deal with COVID-19	PhD in epidemiology

**Table 2:** Themes and subthemes extracted from content analysis

Themes	Subthemes
Clinical capacity improvement	♣ Performance improvement ♣ Logistic improvement
Resource and infrastructure management	♣ Supply and support of human resources ♣ Infrastructure improvement ♣ Supply of equipment
Epidemiology capacity increase	♣ Epidemiology improvement ♣ Emerging disease surveillance
Applying principles of disaster and emergency management	♣ intra- and extra-organizational interaction management ♣ Disaster risk management ♣ Data management
Society resilience increase	♣ Improving adaptation skill and maintaining health ♣ Social participation

### Clinical capacity improvement

One of the categories extracted based on the views of the participants was the clinical capacity improvement of the health system which included two subcategories of performance improvement and logistic improvement.

#### Performance improvement

Based on the views of the participants, the health system preparedness to newly emerging respiratory viruses depends on performance improvement. In this regard, the participants referred to principles such as respiratory triage and isolation in wards and ambulances, correct and scientific use of herbal and traditional medicines, and planning the treatment of patients in a multidisciplinary manner.

According to one of the participants:

*“A major challenge during the crisis of the COVID-19 pandemic, and previously the H1N1 flu epidemic, was the lack of instruction for prioritizing infected people. The hospital emergency personnel were confused because all the patients referred to the hospital emergency room were triaged in the same place and in the same way and there was no specific setting for the triage of patients with COVID-19.” (Participant 14)*

Another participant stated that:

*“To manage a newly emerging respiratory disease that is highly contagious and can have high lethality and serious genetic mutation, a complete team including health and medical fields is needed.” (Participant 9)*

#### Logistic improvement

Another sub-theme of clinical capacity improvement theme was a logistic improvement. According to the participants, the health system should accommodate appropriate clinical and therapeutic services. Preparedness in cases such as vaccine, drug management, management of the transfer of virus-infected patients between medical centers, preparation of special resuscitation teams for newly emerging respiratory diseases, and hygienic transference and burial of the bodies of infected people were emphasized by the participants.

According to one of the participants:

*“At the beginning of the COVID-19 pandemic, private ambulances, which were not supervised, transferred infected patients between cities and hospitals. With the same ambulances, non-COVID-19 patients were also transferred. This caused the spread of the disease and led to dissatisfaction. We really have to plan in advance.“ (Participant 3)*

Another participant said:

*“As for the COVID-19 or any other unknown emerging disease, there is a possibility of transmission from the body, and it is essential to disinfect the body and transfer or bury it safely.” (Participant 6)*

### Resource and infrastructure management

Another theme in this study was resource and infrastructure management. According to the views of the participants, a major element of preparedness for epidemic management is resource and infrastructure management and optimal use. This theme includes sub-themes of provision and support of human resources, infrastructure improvement, and supply of equipment.

#### Provision and support of human resources

According to the participants in this study, providing the necessary human resources, improving the knowledge and skills of the staff, and providing financial, spiritual, and psychological support for them are essential to respond to epidemics. Welfare of the healthcare team during an outbreak should be considered. All of these should be planned before the outbreak occurs. According to one of the participants:

*“To respond to the epidemic, when long-term burnout occurs, there should be a coherent and efficient organization to employ and replace human resources. Also, more welfare facilities should be provided for the personnel and their families who are on the frontline. However, it should be mentioned that doctors and healthcare providers sacrifice was really admirable during COVID-19 epidemic.” (Participant 1)*

Another participant stated that:

*“One part of preparedness to any disaster is the preparedness of the personnel. Their information should be up to date, and they should be mentally and psychologically ready. Therefore, the managers of the health system should make special arrangements for disaster management courses and maneuvers to empower the personnel and hold psychology courses such as resilience and stress management.” (Participant 11)*

#### Resource and infrastructure management

According to the views of the participants, the infrastructure improvement of the health system should respond to newly emerging respiratory viruses. Stablishing special diagnostic laboratories, isolation, quarantine and special care departments, spaces for admitting and keeping the infected people and using new technologies were among the most important points the participants mentioned in this field.

According to one of the participants:

*“For preparedness to respond to an epidemic, we should look into the condition of the spaces and beds that are available for care and treatment at our disposal, and bed and space management should be one of the priorities. It is imperative to pay attention to the preparedness of special care units to respond to new outbreaks.” (Participant 7)*

Another participant stated that:

*“A major shortcoming in responding to the COVID-19 pandemic is the limited use of new technologies. Unfortunately, we still use the methods of forty years ago.” (Participant 12)*

#### Provision of equipment

According to the views of the participants, provision of the required equipment is vital to respond to emerging infectious disease outbreaks. Indispensable equipment that was emphasized by them were specialized medical and care-therapeutic equipment (e.g., oxygen generator and ventilator), personal protective equipment, hand disinfection system and waste disposal equipment.

According to one of the participants:

*“For management of the COVID-19 pandemic, the lack of equipment, especially personal protective equipment, such as gloves and masks, was our serious challenge. The supply and distribution system of the equipment was not suitable, i.e., the supply was low, and the demand was high. I wish they had thought about this in advance.” (Participant 16).*

### Epidemiology capacity increase

A theme emerged in this study was epidemiology capacity, which includes two sub-themes: epidemiology capacity improvement and surveillance of emerging diseases. The main objective of emerging respiratory diseases management is their timely identification and control which is achievable through epidemiology capacity promotion.

#### Epidemiological improvement

According to the views of the participants, the health system should improve its epidemiology capacity to be able to respond to emerging diseases. For this purpose, it should examine the epidemiological situation of other infectious diseases as a preparation measure.

According to one of the participants:

*“To prepare for any new disease, we should understand its epidemiological nature, i.e., the power and manner of contagion and the extent of lethality and emergency, and plan accordingly. It is better to have a center for newly emerging respiratory viral diseases for epidemiologists in the Ministry of Health to communicate with other countries and internal organizations and perform the necessary measures in a concentrated manner.” (Participant 18)*

#### Surveillance of emerging diseases

According to the views of the participants, new respiratory viral diseases need careful surveillance. The health system should be able to monitor the emergence of these diseases and, considering the unknown nature of newly emerging viruses, plan for different types of these viruses, so that they can identify and report the spread of these diseases based on prediction models and respond on time and appropriately. According to one of the participants:

*“In my opinion, the most important challenge in the response to COVID-19 was the lack of recognition of the disease. We did not know the virus itself, its nature, and its behavior, and it was demanding to discover the nature of the disease. We were fighting an unknown enemy.” (Participant 8)*

### Application of the principles of disaster and emergency management

Another theme extracted based on the views of the participants was the application of the principles of disaster and emergency management, which included three sub-themes: intra- and extra-organizational interaction management, disaster risk management, and data management.

#### Intra- and extra-organizational interaction management

The participants mentioned the lack of intra- and extra-organizational interaction management to respond to emerging respiratory viruses in Iran. According to the results, principles such as cooperation and communication within and outside the organization and coordination between different organizations are an integral part of appropriate and effective response to health crises. According to one of the participants:

*“According to the World Health Organization, the disease is controlled by governments, not by a ministry. This shows how important inter-sectoral coordination is, as it can ensure national trust.” (Participant 5)*

Another participant stated that:

*“Unfortunately, regarding COVID-19, there was no unified commander, there was no coherent inter-, intra-, and extra-organizational coordination, there was no proper public relations and it was not even thought about in advance, as if everyone had become their own boss and made their own arrangements. Responding to similar disasters in future is impossible without solving these problems.” (Participant 4)*

#### Disaster risk management

Most participants emphasized the application of the principles of disaster risk management to prepare to respond to the epidemic of emerging respiratory viruses. Although some of them did not directly refer to this, all the participants referred to principles such as risk assessment, unified commander, exercise and maneuver and contingency plan. According to one of the participants:

*“The personnel of the health system, who work in hospitals and health centers, did not participate in the exercise and maneuver that measured their preparedness before the outbreak of the COVID-19 pandemic, and they were not held at all*” *(Participant 2).*

Another participant stated that:

*“Look, my first suggestion for preparedness is to learn from the past lessons. As the Persian saying goes, a believer is* not stung twice *(by something) out of the* same hole. *Well, I have to see what conditions should arise not to be damaged as in previous epidemics such as influenza and COVID-19, so that we will not be harmed in the event of similar events in the future.”* (*Participant 3)*

#### Data management

According to the views of the participants, data management includes collection, analysis, and communication of substantial data to provide an appropriate and effective response to the epidemic of newly emerging respiratory viruses.

In this regard, participant 13 mentioned:

*“In response to COVID-19, our data recording might have had deficiencies and we even did not think about the nature of the data needed. Storing and coding of the data were incorrect, as well. All these are major problems in our data analysis. Otherwise, I believe that if we can analyze the data correctly, we can solve many of our problems and do proper scientific work.”* (*Participant 13)*

### Resilience of the society

Another theme of health system preparedness to respond to emerging respiratory viruses was a boost in resilience, which included two sub-themes: improving adaptation skills and maintaining health and social participation.

#### Improving adaptation skills and maintaining health

The participants acknowledged the decisive role of the adaptation skills of the society in the success of the health system plan to respond to the epidemic of emerging respiratory viruses. According to the views of the participants, the health system should develop skills through training and culture, public trust in the data and actions of the health system, and understanding the structure of the society. In this regard, participant 12 mentioned:

*“If the Ministry of Health develops a plan to inform the public about newly emerging viral diseases, and then train its own personnel in this regard, the personnel convey it to the people and society through the training of health ambassadors, so that if the health literacy of society increases, in case of an epidemic caused by these viruses, people will help us more.” (Participant 12)*

One of the participants stated that:

*“How deeply people trust our cultivation is a significant issue. People will lose trust if they are faced with contradictory remarks and policies. When we see a specialist in infectious diseases ward with no mask, how can we expect people to trust us and follow the instructions?” (Participant 10)*

#### Society participation

The participants mentioned society participation as an essential principle of an appropriate and successful response to emerging respiratory viruses. The summary of their views in this regard included management of the rumors and cyberspace, management of volunteers and benefactors, and monitoring of the compliance of society with health protocols.

Participant 6 asserted:

*“Health measures are considered social rights, and its implementation requires complete participation of the public. The instructions sent to us by the World Health Organization stated: You cannot be successful in a situation that conflicts with the culture of the people. Therefore, to respond to epidemics, we should plan according to people’s culture.” (Participant 6).*

One of the participants stated that:

*“One of our problems in convincing people to get vaccinated during the COVID-19 pandemic was that the groups against vaccination had access to people through cyberspace; on the other hand, because people had access to such data, they could not distinguish between the right and wrong data and accepted the wrong information. This issue shows the importance of planning for management of the cyberspace in similar cases in the future.” (Participant 14).*

## Discussion

In recent years, the health system in Iran has faced many epidemics related to novel respiratory viruses such as H1N1 flu and recently COVID-19. According to the views of the participants, the lack of preparedness of the health system in Iran to respond to these epidemics, especially in the early days of exposure to COVID-19, was evident, and this lack of preparedness was associated with an increase in morbidity and mortality. However, the COVID-19 pandemic revealed the lack of preparedness of the health system around the world, even in economically and medically advanced countries [4, 5]. Therefore, the health system should be ready to respond to possible epidemics in the future. In this regard, the key issues and different fields of health system preparedness to respond to emerging disease outbreaks in the future were identified through this study.

The opinions of the participants indicate the need to improve the clinical capacity of the health system in terms of performance and support, for example, planning for the treatment of patients in a multidisciplinary manner or the implementation of respiratory triage; organizing the admission, hospitalization, and treatment of non-contagious patients; and preparing for appropriate supply and distribution of drugs and vaccines. Management of elective patients can increase the clinical capacity of hospitals to admit and treat the patients infected with respiratory viruses such as COVID-19. This group of patients should be classified based on the urgency of the treatment; however, this issue can negatively impact their health due to the delay in receiving their treatment [15–17]. In this regard, Anesi et al. have emphasized the design and application of clinical protocols and planning and implementing specific triage for patients with emerging infections [17]. In fact, these studies emphasize on the cases whose development is in line with the development of clinical ability, which has been the focus of the participants in the present study.

According to the results of the present study, the management of resources and infrastructures can effectively prepare the authorities to respond to disasters, including pandemics caused by emerging viruses. Lack of qualified and skilled human resources, sufficient space and personal protective equipment can be challenging in response operations. For example, severe stress and fear felt by staff to care for patients with COVID-19 and the problems of personnel families and the restrictions created to be with the family were among the major challenges of responding to COVID-19. Similarly, the problems related to the provision of sufficient and skilled and trained human resources, as well as their protection and support during pandemics caused by emerging respiratory viruses and the need for preparedness in this field have been raised in Canada [16–18]. Therefore, the health system should have a proper plan to train human resources and support them and their families. Yousefi et al. (2022) have also emphasized that the policymakers of human resources in the health system should consider a comprehensive road map for hiring, training, and developing the performance of the personnel for possible future epidemics [8]. Given the type of activity of hospitals, preventing the intra-hospital transmission of emerging virus that can affect the personnel and patients is vital during epidemics. For this purpose, it is necessary to isolate the confirmed and suspected infected people from other patients in special wards and comply with infection control protocols, which requires additional space suitable for hospitalization and isolation and quarantine of patients [19]. Kain and colleagues have also mentioned that since respiratory infections can cause severe involvement of the respiratory system of patients, the health system should have special care units available in cases of epidemics [20]. The study results on the COVID-19 pandemic showed solutions such as closing some wards like surgery or internal medicine through reducing elective surgeries or discharging the patients to provide inpatient space for people infected with COVID-19 who needed hospitalization and using other wards by placing ventilators and basic intensive care equipment in the intensive care unit. However, places such as stadiums and hotels were used to keep people who did not have severe problems and only needed basic measures such as oxygen administration. It should be noted that it was difficult to conclude a contract and convince the relevant managers to use these places as patient care centers, so the necessary memorandums should be written in the preparedness phase. On the other hand, according to the views of the participants, therapeutic and non-therapeutic equipment such as an oxygen generator or oxygen capsule, personal protective equipment, ventilator and bed, and infectious waste disposal equipment are essential for the health system preparedness to face the epidemic of respiratory viruses. During the COVID-19 pandemic, in some countries, due to the lack of personal protective equipment, personnel had to wash and reuse disposable personal protective equipment (16). In a study by Bijani et al. (2021), the lack of medical equipment and drugs has been mentioned as a major challenge for managers for the management of COVID-19 in Iran, and they emphasized the necessity of health system preparedness in this field [21].

As a result, the findings of the present study on the preparation of resources and infrastructures are in line with the findings of other studies, and indicate the importance of the special attention of health managers to this field in order to suitable management of future outbreaks.

According to the results of the present study, the health system should pay special attention to epidemiology; to respond to newly emerging respiratory viruses, the epidemiologic capacity should be increased through research. Moreover, since infectious agents can spread worldwide easily, the emergence of newly emerging infectious diseases should be monitored globally, which needs a robust international epidemiological framework. To this end, a global surveillance system should be established by WHO, and any sign and symptoms of infectious diseases that looks uncommon should be reported promptly to a center in WHO from any point in the world. Also, given the unknown nature of emerging viruses, planning should be done to respond to different types of emerging viruses. In addition, diagnosing and tracking infected and suspicious people and setting up a new respiratory disease center in the Ministry of Health, treatment and Medicine Education must be prioritized by the health system authorities. In confirmation of this issue, Dikid et al. also introduced increasing the epidemiology capacity, monitoring and rapid response mechanism, and conducting research as important principles to improve the capacity of the health system to deal with emerging infectious diseases [22].

Most of the participants agreed on applying the principles of disaster and emergency management to respond to the epidemic caused by emerging respiratory viruses. From their point of view, intra- and extra-organizational cooperation and coordination, risk assessment, exercise and maneuver, unified commander, overcapacity plans, and correct management of data are part of the principles that should be considered to properly manage the crisis caused by emerging respiratory viruses by the health system. However, during the epidemics in Iran, such as the flu epidemics and different peaks of COVID-19, it was observed that traditional management and the principles of disaster management were not exercised in the preparedness and response phase; this can be rooted in the health managers’ lack of knowledge about these principles. This reveals the importance of training in this field for experts. In Iran, measures have been taken to institutionalize these principles and coordinate various organizations to respond to disasters, including holding PhD programs in health in disasters and emergencies and drafting laws for the coordination between organizations through crisis management in the country. However, the lack of basic disaster management and inter- and even intra-sectoral coordination are among the main problems of disaster management in Iran, which is clearly visible in the management of the COVID-19 pandemic [21]. Raofi et al. (2020) also mentioned the lack of decisive and unified management as a challenge in managing COVID-19 in Iran and considered the need for the efforts of all organizations and unified management to properly respond to such conditions [23]. According to a study by Heslop (2020), countries should be ready for unknown and unpredictable infectious diseases based on the principles of risk assessment and design programs that put them at a level of preparedness and planning to be able to manage new infections in the future without surprising the health system and other social resources [24].

The results of the present study showed the essential role of social resilience to respond to emerging respiratory viruses. According to the participant’s views, the health system should increase people’s participation to respond to the outbreaks caused by the newly emerging respiratory virus and resilience, before an epidemic occurs, by training and increasing the public trust in information and measures of the health system and understanding the structures of the society, the skills of adaptation and maintenance of health in the society, which is one of the principles of understanding risk and resilience. In addition, planning to manage the cyberspace, health volunteers and donors, and health protocols, according to the participant’s views, cannot be achieved when health measures are in conflict with people’s culture. Wang et al. also have emphasized strengthening public participation to respond to public health emergencies as a viable solution to respond to the epidemic of emerging viruses [25]. On the other hand, people’s behavior and response to risks are reflection of risk assessment. In this regard, improvement of the five fields in society including culture, public, policy, knowledge, and emotions can improve the culture at individual and societal levels. For this reason, strengthening the cultural, religious, and political structure will enhance people’s compliance with health protocols [26]. It means that in order to enhancing population participation in outbreak management, health managers should be familiar with cultural, social, economic, political, and religious structure of the society and consider these structures in management related activities.

### Limitations

In this study, the view of the senior managers was investigated, while the view of other employees of the health system can also clarify some points in this field. Therefore, it is suggested to investigate the views and experiences of other employees in future studies. As only semi-structured interview was used in this study, it is suggested that other data collection tools should be used in future qualitative studies.

### Strengths

To the best of our knowledge, this is the first qualitative study which aimed to explain the preparedness of the health system against emerging respiratory viruses in Iran. Managers at different management levels and experts from different parts of Iran participated in this study.

## Conclusion

Novel and emerging infectious agents have caused several outbreaks during recent years. On the other hand, as technology develops, viruses change their nature too; therefore, the world will face other outbreaks and pandemic in future. This fact illustrates the necessity of health systems preparedness for future. The results of this study present the key issues for future preparedness. Health system managers and policymakers in Iran and all countries in the world should be aware of these key issues and apply them in practice to prepare the health systems to respond to next outbreaks. Indeed, the study results can help policymakers and health system managers to plan to achieve acceptable preparedness for management of such outbreaks.

## Data Availability

The datasets generated and/or analysed during the current study are not publicly available due to the necessity to ensure participant confidentiality policies and laws of the country but are available from the corresponding author on reasonable request.

## References

[1] Carrasco-Hernandez R, Jácome R, López Vidal Y, Ponce de León S (2017). Are RNA viruses candidate agents for the next global pandemic? A review. ILAR J.

[2] Organization WH. Pandemic influenza preparedness framework: biennial progress report: 1 January 2018-31 December 2019. JSTOR; 2020. p. 9240008683. Report No.

[3] Nisii C, Castilletti C, Di Caro A, Capobianchi M, Brown D, Lloyd G (2009). The european network of Biosafety-Level-4 laboratories: enhancing european preparedness for new health threats. Clin Microbiol Infect.

[4] Devi K. Corona Pandemic: A Lesson For Preparedness in Future. 2020.

[5] Yang X, Yu Y, Xu J, Shu H, Liu H, Wu Y (2020). Clinical course and outcomes of critically ill patients with SARS-CoV-2 pneumonia in Wuhan, China: a single-centered, retrospective, observational study. The Lancet Respiratory Medicine.

[6] Sundararaman T, Muraleedharan V, Ranjan A (2021). Pandemic resilience and health systems preparedness: lessons from COVID-19 for the twenty-first century. J Soc Econ Dev.

[7] Nacoti M, Ciocca A, Giupponi A, Brambillasca P, Lussana F, Pisano M et al. At the epicenter of the Covid-19 pandemic and humanitarian crises in Italy: changing perspectives on preparation and mitigation. NEJM Catalyst innovations in care delivery. 2020;1(2).

[8] Yusefi AR, Sharifi M, Nasabi Ns, Rezabeigi Davarani E, Bastani P (2022). Health human resources challenges during COVID-19 pandemic; evidence of a qualitative study in a developing country. PLoS ONE.

[9] Peleg K, Bodas M, Hertelendy AJ, Kirsch TD. The COVID-19 pandemic challenge to the All-Hazards Approach for disaster planning. Int J Disaster Risk Reduct. 2021.

[10] Hammarberg K, Kirkman M, de Lacey S (2016). Qualitative research methods: when to use them and how to judge them. Hum Reprod.

[11] Holley RP. Applications of social research methods to questions in information and library science. portal: Libraries and the Academy. 2009;9(4):517-8.

[12] Ritchie J, Lewis J, Nicholls CM, Ormston R (2013). Qualitative research practice: a guide for social science students.

[13] Graneheim UH, Lundman B (2004). Qualitative content analysis in nursing research: concepts, procedures and measures to achieve trustworthiness. Nurse Educ Today.

[14] Speziale HS, Streubert HJ, Carpenter DR. Qualitative research in nursing: advancing the humanistic imperative. Lippincott Williams & Wilkins; 2011.

[15] Wurmb T, Scholtes K, Kolibay F, Schorscher N, Ertl G, Ernestus R-I (2020). Hospital preparedness for mass critical care during SARS-CoV-2 pandemic. Crit Care.

[16] Alami H, Lehoux P, Fleet R, Fortin J-P, Liu J, Attieh R (2021). How can health systems better prepare for the next pandemic? Lessons learned from the management of COVID-19 in Quebec (Canada). Front Public Health.

[17] Anesi GL, Lynch Y, Evans L. A conceptual and adaptable approach to hospital preparedness for acute surge events due to emerging infectious diseases. Crit care explorations. 2020;2(4). 10.1097/CCE.0000000000000110 PMC7188427 32426752

[18] Guyon Ai, Hancock T, Kirk M, MacDonald M, Neudorf C, Sutcliffe P (2017). The weakening of public health: a threat to population health and health care system sustainability. Can J Public Health.

[19] Wurmb T, Scholtes K, Kolibay F, Schorscher N, Ertl G, Ernestus R-I (2020). Hospital preparedness for mass critical care during SARS-CoV-2 pandemic. Crit Care.

[20] Kain T, Fowler R (2019). Preparing intensive care for the next pandemic influenza. Crit Care.

[21] Bijani M, Karimi S, Khaleghi A, Gholampoor Y, Fereidouni Z (2021). Exploring senior managers’ perceptions of the COVID-19 Crisis in Iran: a qualitative content analysis study. BMC Health Serv Res.

[22] Dikid T, Jain SK, Sharma A, Kumar A, Narain JP (2013). Emerging & re-emerging infections in India: an overview. Indian J Med Res.

[23] Raoofi A, Takian A, Sari AA, Olyaeemanesh A, Haghighi H, Aarabi M. COVID-19 pandemic and comparative health policy learning in Iran. Archives of Iranian Medicine (AIM). 2020;23(4). 10.34172/aim.2020.02 32271594

[24] Heslop DJ (2020). Disaster preparedness to exotic and emerging infections. Microbiol Australia.

[25] Wang J, Wang Z (2020). Strengths, weaknesses, opportunities and threats (SWOT) analysis of China’s prevention and control strategy for the COVID-19 epidemic. Int J Environ Res Public Health.

[26] Ezat S, Fatemeh G, Mina N, MohammadHassan R. Perception risk, preventive behaviors and assessing the relationship between their various dimensions: a cross-sectional study in the Covid-19 peak period. Int J Disaster Risk Reduct. 2022:103093. 10.1016/j.ijdrr.2022.103093 PMC9174951 35694686

